# The Relationship between Modified Short Physical Performance Battery and Falls: A Cross-Sectional Study of Older Outpatients

**DOI:** 10.3390/geriatrics6040106

**Published:** 2021-10-30

**Authors:** Kazuki Fukui, Noriaki Maeda, Makoto Komiya, Junpei Sasadai, Tsubasa Tashiro, Mitsuhiro Yoshimi, Shogo Tsutsumi, Satoshi Arima, Kazuki Kaneda, Satoshi Onoue, Toshiya Shima, Manabu Niitani, Yukio Urabe

**Affiliations:** 1 Graduate School of Biomedical and Health Sciences, Hiroshima University, Hiroshima 734-8553, Japan; kazuki-fukui@hiroshima-u.ac.jp (K.F.); norimmi@hiroshima-u.ac.jp (N.M.); makoto-komiya@hiroshima-u.ac.jp (M.K.); tsubasatashiro716@hiroshima-u.ac.jp (T.T.); mitsuhiroyoshimi0116@hiroshima-u.ac.jp (M.Y.); shogo-tutumi@hiroshima-u.ac.jp (S.T.); satoshi-arima4646@hiroshima-u.ac.jp (S.A.); kazuki-kaneda@hiroshima-u.ac.jp (K.K.); 2 Sports Medical Center, Japan Institute of Sports Sciences, Japan Sport Council, Tokyo 115-0056, Japan; jumpei.sasadai@jpnsport.go.jp; 3 Niitani Clinic, 2-8-18 Nakadori, Kure, Hiroshima 737-0046, Japan; g210119@hiroshima-u.ac.jp (S.O.); toshiyashima@niitani-clinic.jp (T.S.); info@niitani-clinic.jp (M.N.)

**Keywords:** aged, accidental falls, short physical performance battery, physical function performance, logistic models

## Abstract

The Short Physical Performance Battery (SPPB) is a physical fall-risk screening tool and predictor of adverse health effects for the older. Its limited use in Japan is due to the relative ease for high-functioning older adults to achieve perfect scores. Japanese researchers thus created a community-based SPPB (SPPB-com). This study investigated whether the SPPB-com score can distinguish between older patients classified as “fallers” and “non-fallers.” Participants comprised 185 older outpatients aged 65 and above who self-reported their history of accidental falls and relevant physical activity. Fall risk was assessed using SPPB and SPPB-com. Handgrip strength, maximum isometric knee extensor strength, and maximum walking speed were measured as physical functions. Multivariate logistic regression and receiver-operating characteristic analyses determined criteria indicating faller status. Fallers were older and had lower physical function, physical activity, SPPB, and SPPB-com scores than non-fallers. Multivariate logistic regression analysis showed SPPB (OR 0.76, 95% CI 0.59–0.99, *p* = 0.045) and SPPB-com (OR 0.63, 95% CI 0.45–0.87, *p* = 0.005) scores were both independently associated with prior falls. The SPPB-com score may function as a fall-risk assessment tool for older outpatients, and its combined use with SPPB can increase the accuracy and precision of distinction between fallers and non-fallers.

## 1. Introduction and Background

Among the older, physical falls are harmful events that predict adverse health implications, including disability and death [1]. The most harmful consequences of falls are hip fractures and brain damage, although even physically harmless falls are associated with anxiety, depression, and decreased mobility, which greatly affect quality of life (QOL) and aging process trajectory [2]. As physical activities and QOL for older adults are easily affected by fall-related injuries, rehabilitation therapy should aim to prevent these falls. A recent systematic review and meta-analysis emphasized the importance of exercise combined with multidimensional clinical and environmental interventions for fall prevention [3]. Although various fall prevention measures exist, a more accurate assessment of individuals and multifaceted interventions based on these revised assessments are essential [4]. Fall-risk research is becoming increasingly important to maintain healthy lifestyles in the older population.

Several relevant measurements have been described and validated. Some balance-evaluating tools, such as the Berg Balance Scale, exhibit fairly good sensitivity and specificity in predicting falls [5,6] but are difficult to measure and time-consuming, thus restricting their feasible application in everyday clinical practice. Therefore, for older outpatients, fall-risk assessment generally relies on simpler tools.

The Short Physical Performance Battery (SPPB) [7] has been used to predict adverse health effects and as a fall-risk screening tool for the aged [8]. This measurement is widely used in various studies investigating older people’s physical abilities, especially complex abilities, including lower limb muscle strength, 4 m walking speed (at normal pace), and balance [7]. It can be rapidly executed using simple equipment and is sensitive to functionality changes over time. However, it is not widely used in everyday clinical practice in Japan because it is relatively easy for high-functioning older adults to achieve a perfect score (prejudicing its value as a sensitive indicator of physical performance capability) [9]. Early fall-risk detection and prevention for healthy older people are important, especially among Japan’s aging population. Therefore, this tool needs modifying to effectively assess high-functioning older adults.

Makizako et al. [10] used a community-based scoring system to create a modified test-set, SPPB-com, to accomplish this goal. It utilizes the same tests as SPPB but with a different scoring system (0–10 point score). Makisako et al. [10] conducted a 24-month follow-up study of 4328 community-dwelling older adults to determine the incidence of primary nursing care. The results showed that the incidence of primary nursing care was 2.1% for a score of 12 SPPB (perfect score) and 10.4% for a score of 11 or less. On the other hand, the primary nursing care incidence rate was 1.2% when the SPPB-com score was 8 or higher, 3.5% when the score was 5–7, and 12.8% when the score was 4 or lower. This indicates that SPPB-com can predict the incidence of primary nursing care in more detail than SPPB. Thus, we believe that the SPPB-com is more sensitive than the SPPB score in predicting the incidence of primary nursing care. Although SPPB-com has the potential for predicting fall risk in high-functioning older adults, no research has yet been undertaken to define the specific criteria for fallers.

We therefore hypothesized that SPPB-com would be more accurate and precise in predicting falls of older adults than SPPB. This cross-sectional study aimed to investigate whether the SPPB-com score could effectively distinguish between fallers and non-fallers among older outpatients.

## 2. Methods

### 2.1. Study Design and Subjects

**Figure 1:**
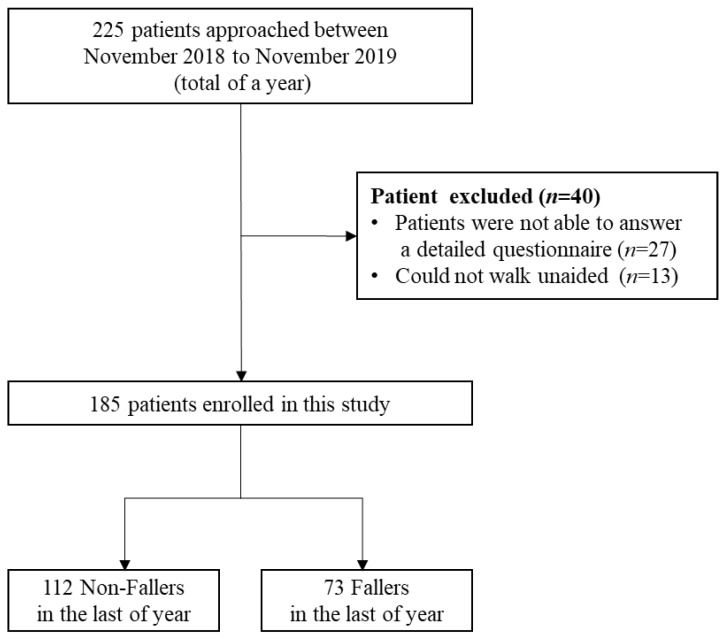
Participant recruitment flow diagram.

The 185 participants were older outpatients aged 65 and older. Participants were registered at an orthopedic clinic in Hiroshima, Japan, during November 2018–November 2019. Patients who had visited this clinic for longer than two months, at least once a week, and who did not have decreased cognitive function were included. Cognitive function was assessed using the revised Hasegawa Dementia Scale, and a score of 21 or higher was considered to indicate no decline in cognitive function [11]. We excluded subjects who were unable to answer a detailed questionnaire or walk unaided (Figure 1). Before the study, all participants provided informed consent for the paper, which was reviewed and approved by the institutional review board of the Niitani Clinic (NCL-18002).

### 2.2. Metrics

#### 2.2.1. Sample Characteristics

We recorded participants’ demographic characteristics, including age, sex, height, weight, body mass index, fall history, and physical activity level—as measured by the self-administered Physical Activity Questionnaire for Elderly Japanese (PAQ-EJ) [12], which covers typical activity patterns of daily life. We then converted this score into metabolic equivalents of task (MET) hours per week (MET h/week). These assessments were also self-reported.

#### 2.2.2. Measuring Fall Experience

Participants were defined as fallers or non-fallers based on their self-reported fall experiences in the past year. A “history of falls” was defined as “unintentionally coming to the ground or a lower level as a consequence of sustaining a violent blow, loss of consciousness, or sudden onset of paralysis as in stroke or an epileptic seizure” [13].

##### Outcome Measures—Muscle Strength

Handgrip strength and maximum isometric knee extension strength were regarded as muscle strength indicators by three physical therapists. Handgrip strength was tested twice with the dominant hand using a digital dynamometer (T.K.K.5401, Takei Kiki Kogyo, Japan); the stronger test result was used in the analysis [14].

The maximum isometric knee extensor strength was assessed using a handheld dynamometer (Mobie, Sakai Med Co., Tokyo, Japan) when participants were seated on a treatment table with their knees and hips at 90° flexion. The sensor pad was fixed to the distal lower leg with a Velcro band and connected to the posterior lower leg brace, and the distal lower leg with a belt. Next, the length of the belt was connected to the lower leg. A folded bath towel was placed under the knee to avoid pain from pressure on the knee fossa during the measurement. The trunk was supported in a vertical position, and both upper limbs were crossed anterior to the trunk.

Participants were then instructed to perform an isometric knee extension exercise with maximum effort for approximately three seconds, in which the maximum value was recorded. The average of each participant’s highest of two recordings from each side was used [15].

##### Outcome Measures—Gait Ability

Maximum walking speed was measured over 10 m. Participants were instructed to walk as quickly as possible from one meter before the starting line until one meter after the finish line. The timing commenced when the participant’s swing leg crossed the start line and ended when the swing leg passed over the finish line. The shortest time from two trials was used for calculations [16].

#### 2.2.3. Short Physical Performance Battery

Both SPPB (0–12 points) and SPPB-com (0–10 points) were measured as physical performance. SPPB comprises three assessments:

1. A balance test;
2. A 4 m walk test; and
3. A repeated chair stand test.

Each SPPB component (balance, walk, and chair stand) was marked from 0 (inability to perform the test) to 4 (best possible performance). For the balance test, participants were asked to keep their feet in a side-by-side position, then in a semi-tandem position, and finally in a fully tandem position, each for 10 s. For the 4 m walk test, participants walked at their usual speed. For the repeated chair stand test, subjects were asked to stand up and sit down five times as quickly as possible. This task was only conducted once. The SPPB score was calculated according to the literature’s prescribed standards [7].

The SPPB-com tests were conducted in a similar manner to SPPB but scored differently. The score was calculated following Makizako et al. [10]. For the balance test, participants unable to remain in a tandem stance received 0 points; those who maintained this position for 1–10 s earned 1 point; and >10 s earned 2 points. For the 4 m walk test, participants who could not walk four meters received 0 points; those who completed it in ≤1.10 m/s, 1.11–1.24 m/s, 1.25–1.36 m/s, and ≥1.37 m/s received 1, 2, 3, and 4 points, respectively. Participants who could not complete the chair stand five times received 0 points; those who completed it in ≥9.70 s, 8.15–9.69 s, 6.85–8.14 s, and ≤6.84 s earned 1, 2, 3, and 4 points, respectively. The highest possible overall score was 10 points (Table 1).

### 2.3. Statistical Analysis

Continuous variables were reported as mean ± standard deviation (SD) or as median and interquartile range (IQR), according to the normality of the distribution of values. All population characteristics were stratified according to participants’ self-reported fall history and were compared between groups using the *t*-test, Mann–Whitney U test, or chi-square test. An analysis of the receiver-operating characteristics (ROC) of the SPPB scale and the SPPB-com scale was conducted by calculating the area under the curve (AUC) and the cutoff point. This served to better distinguish fallers from non-fallers. History of falls was subsequently tested using multivariate logistic regression models, considering the SPPB and SPPB-com scores. The model was simplified in a backward stepwise (Wald) method by removing variables with *p*-values > 0.05. The goodness of fit of the models was assessed with the omnibus test of model coefficients and the Hosmer–Lemeshow test. Covariates for the multivariate analysis were selected, considering the variables with a significant difference between fallers and non-fallers at preliminary comparisons. Statistical analyses were conducted using SPSS for Windows v.25.0 (IBM SPSS Inc., Tokyo, Japan), with a significance threshold of 0.05.

## 3. Results

Participants’ demographic data, stratified by fall history in the preceding year, are shown in Table 2. The number of fallers—subjects reporting ≥1 fall in the past year—was 73 (39.5%); the number of non-fallers was 112 (60.5%).

Table 2 also compares the principal variables between fallers and non-fallers. Fallers were significantly older than non-fallers (*p* = 0.005). Relative to non-fallers, fallers also displayed significantly lower levels of handgrip strength, knee extensor strength, and maximum walking speed, and lower scores on PAQ-EJ, SPPB, and SPPB-com. Furthermore, 102 participants (55.1%) received a perfect score on SPPB, whereas only 3 (1.6%) scored perfectly on SPPB-com.

**Figure 2:**
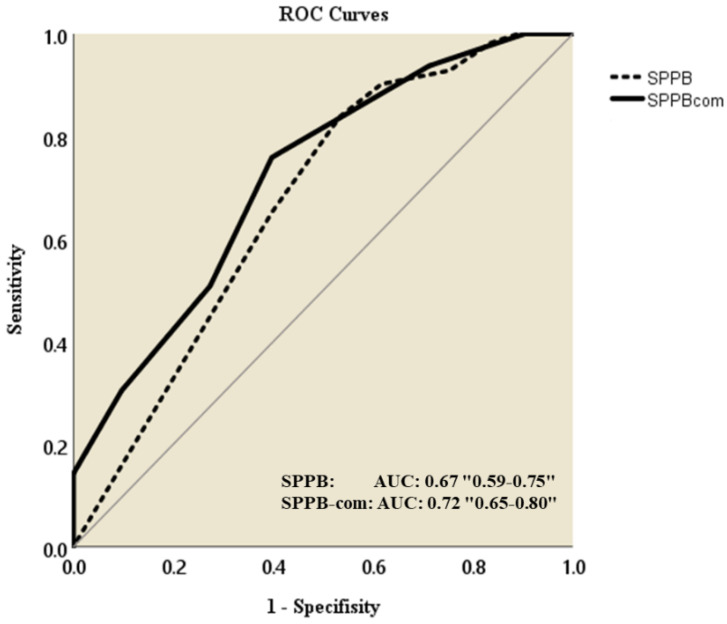
ROC curves for SPPB and SPPB-com to predict fallers. SPPB: Short Physical Performance Battery, SPPB-com: Short Physical Performance Battery-community based.

Table 3 and Figure 2 presents the ROC analysis aimed at predicting fallers. The score giving the best trade-off between sensitivity and specificity is 11 on SPPB (AUC = 0.67) but 5 on SPPB-com (AUC = 0.72).

In the multivariate logistic regressions (Table 4), considering a long list of possible covariates, both the SPPB score (Model 1: OR 0.76, 95% CI 0.59–1.00., *p* = 0.046) and the SPPB-com score (Model 2: OR 0.63, 95% CI 0.45–0.87, *p* = 0.005) were negatively associated with a history of falls. There was no suspicion of multicollinearity among all items included in the multivariate logistic regressions.

## 4. Discussion

Our most important finding was that the SPPB-com score can effectively determine the difference between fallers and non-fallers in older outpatients. Our results indicated that the SPPB and SPPB-com scores were significantly associated with a history of falls. SPPB-com, specifically modified to assess high-functioning older adults (such as Japanese community-dwellers), matched SPPB in discriminating between fallers and non-fallers.

SPPB comprises three assessments of multiple physical performance factors: muscle strength, balance, and gait ability. Our study’s fallers group had reduced muscle strength, walking speed, and physical activity compared with non-fallers. The results of multiple logistic regressions show the association between the SPPB score and fall history (Table 4, Model 1). Previously, the SPPB score was significantly associated with the frailty index and increased fall risk in a large population-based study [17]. Prior studies comparing SPPB with other fall assessment tools have demonstrated the same relationship between SPPB and falls as our study [12]. Therefore, our findings indicate that SPPB is an effective fall-risk assessment tool for distinguishing fallers and non-fallers.

Moreover, the SPPB-com score showed equivalent, if not superior, ability to SPPB in distinguishing fallers and non-fallers. SPPB and SPPB-com use the same measurement rubric, evaluating lower limb abilities in a composite manner. Furthermore, the SPPB-com score had a larger AUC and a higher odds ratio than the SPPB score, which could more reliably identify fall risk in older outpatients (Figure 2; Table 4, Model 2).

The only difference between SPPB and SPPB-com is the score calculation system. The SPPB-com score was applied to high-functioning older adults in Japan [10], which SPPB cannot reliably evaluate [18]. While 55.1% of participants achieved a perfect SPPB score, only 1.6% achieved a perfect SPPB-com score. This result suggests that SPPB-com can readily assess patients who cannot be reliably evaluated by SPPB. Additionally, in the ROC analysis results, the SPPB score had an AUC of 0.67, 84% sensitivity, and 47% specificity, compared to an AUC of 0.72, 76% sensitivity, and 60% specificity for the SPPB-com score. The AUC value of <0.70 suggests that SPPB misclassified many fallers as having a low fall risk. This is consistent with several prior studies indicating that traditional tools have limited capacity to correctly stratify fall risk in older outpatients [5].

Perera et al. [19] reported that because of the integer increments in the SPPB score, even if there was a statistically significant difference (0.5 points), the change could not be captured clinically. Furthermore, in a previous study of a 6-month exercise intervention in the elderly, the mean baseline SPPB score was 10.1 ± 1.5 (range: 7–12 points), and after the intervention, the SPPB improved significantly by 0.6 points; however, there was no significant improvement in the balance test score, a subscale of the SPPB [20]. These results suggest that changes in SPPB scores are often smaller than 1 point on average, and that it is difficult to reflect small changes in physical function in the scores. We needed to examine the effectiveness of these interventions after identifying subjects with a higher fall risk. Even if SPPB alone identifies fall risk, it is not possible to confirm whether the effect of the intervention has been obtained since then. However, when SPPB-com is evaluated together with SPPB, the intervention’s effect can be confirmed from smaller changes. The previous study also suggested that a 1-point decrease in the SPPB-com score reduced the risk of requiring nursing care by 23% [10] and that SPPB-com could more reliably evaluate exercise intervention effects.

Fall risk may depend on not only muscle weakness and sarcopenia but also cognitive impairment and clinical tools. Integrating both aspects of the risk profile may be useful in clinical practice [21]. However, there are time constraints in day-to-day practice, as therapists attend to each patient. Therefore, clinicians require clinical tools that can evaluate older outpatients quickly and accurately. SPPB can be used as a simple and accurate screening tool in combination with SPPB-com.

This study had some limitations. The cross-sectional design prevents inferring the ability of SPPB and SPPB-com to predict falls in long-term follow-up. The association between SPPB and SPPB-com scores and faller status does not necessarily suggest that the fall was caused by poor physical ability: the lower scores among fallers could have resulted from a previous injurious fall or from more cautious movements for fear of falling. Participants’ cognitive level allowed them to respond to the questionnaire alone, and no cognitive decline was apparent. However, cognitive functioning was not specifically investigated. Likewise, due to regional and sex bias among participants, further studies should increase the sample size and examine the accuracy of these criteria.

## 5. Conclusions

This study is the first to describe the discrimination capability of the new SPPB-com criteria for fallers and non-fallers. Our findings demonstrate that, among older outpatients, SPPB-com is not inferior to SPPB for distinguishing fallers and non-fallers. Clinicians who perform fall-risk assessments of older patients should be made aware that SPPB may have a ceiling effect, although one may calculate SPPB-com solely by performing SPPB.

## Figures and Tables

**Table 1:** Scores on the Short Physical Performance Battery (SPPB) and the SPPB-community-based.

SPPB score	Score	Balance test	Repeated chair stands	Gait speed
	0	Impossible or cannot keep 10 s side-by-side	Impossible	Impossible
	1	Keep 10 s side-by-side and keep under 10 s semi-tandem	16.70 s~	~0.43 m/s
	2	Keep 10s semi-tandem and keep 0–2 s tandem	13.70~16.69s	0.44~0.60 m/s
	3	Keep 10 s semi-tandem and keep 3–9 s tandem	11.20~13.69 s	0.61~0.77 m/s
	4	Keep 10 s tandem	~11.19 s	0.78 m/s~
SPPB-community based score	Score	Balance test	Repeated chair stands	Gait speed
	0	Impossible tandem	Impossible	Impossible
	1	Keep 0–10 s tandem	9.70 s~	~1.10 m/s
	2	Keep tandem > 10 s	8.15–9.69 s	1.11–1.24 m/s
	3	-	6.85–8.14 s	1.25–1.36 m/s
	4	-	~6.84 s	1.37 m/s~

**Table 2:** Demographic differences between fallers and non-fallers.

	Total (*n* = 185)	Fallers (*n* = 73)	Non-Fallers (*n* = 112)	* *p* Value (Fallers vs. Non-Fallers)
Sex (male)	48 (25.9%)	18 (24.7%)	30 (26.8%)	0.75
Age (y)	77.6 ± 6.3	79.2 ± 6.2	76.6 ± 6.2	**0.005**
Body mass index (kg/m^2^)	23.8 ± 3.7	23.5 ± 3.6	24.1 ± 3.7	0.31
Grip strength (kg)	20.75 (16.90–26.48)	18.80 (16.30–23.4)	21.90 (17.98–27.18)	**0.01**
Knee extension strength (N/kg)	4.12 (3.23–26.48)	3.56 (2.93–4.53)	4.56 (3.71–5.52)	**<0.001**
Maximum walking speed (m/s)	1.35 ± 0.37	1.21 ± 0.37	1.44 ± 0.34	**<0.001**
PAQ-EJ (score)	48.32 (24.49–74.17)	40.10 (18.68–64.19)	51.84 (31.18–85.98)	**0.008**
SPPB (score)	12 (10–12)	11 (9–12)	12 (11–12)	**<0.001**
SPPB-com (score)	5 (4–6)	4 (3–6)	6 (5–7)	**<0.001**

Significant *p* values (<0.05) are indicated in bold. * *p* value calculated with *t*-test, Mann–Whitney U test, or chi-square test. Data expressed as mean ± SD, median (IQR), or number (%), as appropriate. PAQ-EJ: Physical Activity Questionnaire for Elderly Japanese, SPPB: Short Physical Performance Battery, SPPB-com: Short Physical Performance Battery-community-based.

**Table 3:** Sensitivity and specificity of Short Physical Performance Battery tests based on differentiating between fallers and non-fallers.

Fall	Cutoff Point	Sensitivity (%)	Specificity (%)	AUC (CI 95%)
SPPB	10/11	84	47	0.67 (0.59–0.75)
SPPB-com	4/5	76	60	0.72 (0.65–0.80)

SPPB: Short Physical Performance Battery, SPPB-com: Short Physical Performance Battery-community-based.

**Table 4:** Multivariate logistic regression; association of falls with SPPB score or SPPB-com score.

Variable	Model 1		Model 2	
	OR (95% CI)	*p* Value	OR (95% CI)	*p* Value
SPPB score	0.76 (0.59–0.99)	**0.046**	—	—
SPPB-com score	—	—	0.63 (0.45–0.87)	**0.005**
Sex	1.01 (0.33–3.11)	0.99	1.20 (0.39–3.73)	0.75
Age	1.02 (0.96–1.09)	0.46	1.02 (0.96–1.08)	0.49
Grip strength (kg)	1.02 (0.94–1.10)	0.62	1.02 (0.95–1.10)	0.53
Knee extension strength (N/kg)	0.78 (0.57–1.06)	0.11	1.03 (0.95–1.10)	0.21
Maximum walking speed (m/s)	0.65 (0.16–3.19)	0.65	1.12 (0.23–5.26)	0.89
PAQ-EJ score	1.00 (0.99–1.00)	0.26	1.00 (0.99–1.09)	0.21

Significant *p* values (<0.05) are indicated in bold. PAQ-EJ: Physical Activity Questionnaire for Elderly Japanese, SPPB: Short Physical Performance Battery, SPPB-com: Short Physical Performance Battery-community-based.

## Data Availability

No new data were created or analyzed in this study. Data sharing is not applicable to this article.
